# Models for implementing continuing professional development programs in low-resource countries

**DOI:** 10.15694/mep.2017.000018

**Published:** 2017-02-01

**Authors:** Heather G Mack, Karl C Golnik, Neil Murray, Helena P Filipe

**Affiliations:** 1 University of Melbourne; 2 International Council of Ophthalmology; 3 The Fred Hollows Foundation; 4 International Council of Ophthalmology

**Keywords:** Continuing medical education, Continuing professional development, Twinning, Low-resource

## Abstract

This article was migrated. The article was marked as recommended.

Implementing CPD programs for medical professionals has been demonstrated to improve patient outcomes, and is increasingly required for continuing medical licensure. However, implementing programs in low-resource settings is challenging. This review has identified 5 different models for implementation: 1) twinning, 2) greenfield, 3) formal program guided by international medical bodies, 4) directed by regulators and 5) informal program guided by international medical bodies. The models, advantages and disadvantages and costs are discussed. There is minimal evidence on success of implementation models, and further studies are necessary.

## Introduction

Continuing Professional Development (CPD) is the means by which members of the medical profession maintain, develop, update and enhance their knowledge, skills and performance to ensure that they deliver appropriate and safe care (Medical Board of Australia, 2016). CPD is a documented, practice-based and self-directed process, includes reflective learning, typically surgical audit and development goals, and incorporates both formal and informal learning (Davis *et al*, 2008, Filipe *et al*, 2014). Performing CPD has been shown to be effective in improving physician performance (Mazmanian and Davis, 2002) and patient outcomes, and is related to patient safety (Teasdale, 2002). Demonstration of CPD is increasingly required for revalidation/recertification for maintenance of professional licensure in high resource, and more recently in low resource countries (Miller *et al*, 2008; Archer *et al*, 2015).

Low resource countries typically have limited funds to cover health care costs leading to limited access to equipment, less‐developed infrastructure and fewer trained personnel (Hudspeth *et al*, 2015). These factors result in many additional challenges in establishing CPD programs. Similar challenges exist in implementing CPD in other low-resource settings such as remote and rural locations in developed countries (Estrada *et al*, 2011).

This review has identified five models for implementing CPD programs in low-resource settings: twinning, greenfield, formal programs guided by international medical bodies, directed by regulators and informal programs guided by international medical bodies (Table 1). Other than regulatory directed, the models are not mutually exclusive. The models are discussed in terms of advantages, disadvantages and costs to partner organizations. This review focuses on high level program design and implementation, and excludes methods of CPD material delivery.

## General principles of CPD program implementation in low-resource countries

This is well reviewed by Giri *et al* (2010) who note the low evidence base regarding implementing CPD programs. Their general principles are summarized in Figure 1. Their key observations are that CPD programs and supporting activities will be ineffective if CPD is irrelevant to health workers' practice setting, attended only because participants receive per diem, geared towards improving a skill for which there is no demonstrated need, and taken only to meet regulatory requirements rather than to close a competency gap. An evolving consensus is that adequate internet access is a critical component as well.

## Models for implementing CPD in low-resource countries

1. Twinning

Global health partnerships are a well-established method of improving clinical skills in low resource countries (Crisp, 2007, Collins *et al*, 2015), particularly surgical skills in Africa (Lipnick *et al*, 2013). This model is promoted by the World Health Organization/World Federation of Medical Education (2009). Examples of established global programs include the Medical Education Partnership Initiative ( www.mepinetwork.org) and the Vision2020 Links Programme ( http://iceh.lshtm.ac.uk/vision-2020-links-programme).

Twinning is a specific partnership which directly pairs organizations in low-resource and high-resource countries, and designs and implements projects based on needs identified by the low-resource partner. The first examples of twinning used to design and implement CPD programs are continuing education for nurses in Bangladesh (Tyer-Viola *et a*l, 2013, physicians in Laos (Yoon, *et al* 2016) and ophthalmologists in Cambodia (Mack *et al*, 2017). CPD in Cambodia was implemented through partnership between the Cambodian Ophthalmological Society (COS) and the Royal Australian and New Zealand College of Ophthalmologists (RANZCO). The COS-RANZCO partnership was designed as a two-year project to improve capacity in Cambodia, with the CPD program continuing independently after its completion. Twinning is typically funded by the high-resource country, directly by the high-resource partner medical institution, or indirectly by not-for-profit medical or educational agencies or government bodies. In some cases participants may bear part of the cost, for example Bangladeshi nurses (Tyer-Viola *et al*, 2013).

Advantages of twinning are that the CPD program benefits from the educational strength of the high-resource partner, while being specific for the needs of the low-resource country in which it is being implemented. The cost to the low-resource partner is minimized. The Cambodian CPD program required an unanticipated upgrade of the COS server and employment of an additional staff member, which was borne by the COS and the Australian Government Department of Trade through the East Asia Vision program. The cost to RANZCO, the high-resource partner, has been estimated at about $100,000 AUD over the two-year program period and comprised 0.5 full-time equivalent RANZCO staff member, and teaching trips to Cambodia by the Australian and New Zealand-based faculty on a voluntary basis.

Successful twinning projects require buy-in by the low-resource country partner, and adequate time for capacity building so that the program may continue independently after withdrawal of the high-resource partner, good internet access and finally, but critically, an appropriate regulatory environment. In Cambodia, two years was insufficient time to develop independent capacity, and performance of CPD was not a requirement for ongoing medical licensure. A follow-on period of limited support, likely to be two or more years, and CPD requirement for licensure are key components of a successful and enduring twinning program.

2. Greenfield

A greenfield CPD program begins with a blank sheet of paper. The project is led by a medical professional body. Collaborating with stakeholders, the concept of CPD, the competencies required by professionals in the field, curriculum, plan for required activities and implementation plan are developed. An example is the development of CPD for nurses in Lesotho, directed by national nursing leadership (Moetsana-Poka *et al*, 2014). Although not a low-resource country, the RANZCO CPD program was designed by one of the authors (HGM) in 2008 using a greenfield approach. A greenfield CPD project is typically funded by the low-resource country, although seed funding may be contributed by international agencies.

The main advantage of a greenfield CPD program is that it is specific to the needs of the country in which it is developed. Working closely with stakeholders enables all regulatory hurdles to be met. Disadvantages include the need for high level educational expertise (which the professional body may not have), and relatively high cost to the low-income country. Moetsana-Poka *et al* note that the process needs to be staged and includes constant monitoring, evaluation and revision. Buy-in by individual practitioners is necessary for ongoing program success.

3. Formal program guided by an international medical body

CPD programs designed by international medical bodies are typically based on specialist medical disciplines. The best example is the CPD program designed by the International Council of Ophthalmology (ICO, www.icoph.org) a member-based organization that includes about 100 national ophthalmological societies and 40 international subspecialty societies. The ICO collaborates with the World Health Organization, the International Agency for the Prevention of Blindness and international non-government organizations. A similar program is run by the World Federation of Neurology for Latin America (Medina and Munsat, 2010).

In this model the ICO has developed a curriculum (Zagorski 2006) and recording template for ophthalmology CPD. These are intended to be contextualized for each country in which the CPD program is implemented. CPD program directors are supported by a series of Ophthalmic Educators meetings offered annually in low-resource countries, and by an on-line Center for Ophthalmic Educators (https://educators.icoph.org). A CPD handbook has been developed for program directors and participants and covers concepts of CPD and the perspectives of participants, educators, providers and regulators (Filipe *et al*, 2015). The ICO has also developed content for CPD programs, available on-line and in multiple languages. The ICO is a not-for-profit organization incorporated in Switzerland and is funded by member societies (dues paid weighted by country income), income from running bi-annual World Ophthalmology Congress scientific meetings, and the ICO Foundation. All CPD material is available for free to member societies, with the intention of minimizing cost to low-income country societies using the material.

Advantages of this model are that it can be customized to the needs of the country, that it specifically relates to the competencies required by practitioners in the subspecialty discipline and that the cost to the low-income country is minimized. Extensive support mechanisms are available to CPD program directors. A disadvantage is that implementing the program requires internet access.

4. Directed by regulators

A CPD program directed by regulators is designed and implemented by regulatory bodies. Stakeholders including practicing health professionals, professional societies and university medical schools/medical educators may not be consulted. A good example is mandatory Maintenance of Certification in ophthalmology in the USA, which is effectively directed by the American Board of Ophthalmology (www.abop.org) and the Accreditation Council for Continuing Medical Education (www.accme.org) using the points system of the American Medical Association (American Medical Association, 2010). The US ophthalmology professional society (American Academy of Ophthalmology, www.aao.org) functions to supply ophthalmologists with CPD material, much of which is tailored to the requirements of the regulators. Similarly, UK revalidation, which requires structured CPD activities, has been designed and is implemented by the General Medical Council ( www.gmc-uk.org/doctors/revalidation.asp).

The main advantage of the regulatory model is that the CPD program meets regulatory requirements by definition. Some argue that a regulatory framework increases legitimacy of CPD. Disadvantages include 1) Lack of control by medical professionals. The program may not emphasize key competencies for medical professionals in general, or for specific craft groups such as ophthalmology. 2) Cost. This is likely to be the most expensive model for designing and implementing CPD programs because in many instances it requires infrastructure for one or more regulatory bodies. External funders are unlikely to contribute funds to regulators. 3) Effectiveness. There is no evidence that programs controlled by regulators are more effective than those controlled by medical professionals.4) Risk of excessive and time-consuming bureaucratic processes. For example, in the UK doctors under investigation have been reported to commit suicide due to lack of timely processes by the General Medical Council (Horsfall, 2014). 5) Risk of politicization of CPD. Grant and Zilling (2015) argue that this has already occurred in the European Union (EU) with release of an EU Directive on CPD (2013).

5. Informal program guided by an international medical body

CPD is recognized as having both formal and informal activities such as spontaneous interactions with colleagues and reflections on one's own experiences. Informal internet-based opportunities for learning were described by McNamara *et al* in 2007. A good example of an informal program guided by an international medical body is HIV/AIDS continuing education in Africa created by the Institute of Tropical Medicine, Antwerp, Belgium (Zolfo *et al*, 2006). The Internet-based program aims to improve education by provision of guidelines, case studies, frequently-asked questions, useful links and interactive quizzes (http://telemedicine.itg.be). The program does not have formal recording of activities or assessment, but is driven by user requests for information. Another example is RANZCO hosting on-line ongoing education with the Pacific Eye-Care Society (PacEYES), a regional eyecare professional membership organization in the South Pacific (www.paceyes.org). Consistent informal feedback from PacEYES members speaks to the value of such an informal peer to peer network. Advantages of this model are that it is inexpensive, requiring only internet-access and digital cameras, and able to reach a large number of participants even in very remote areas. Internet based CPD has been shown to be effective, compared to no intervention (Cook *et al*, 2008; Casebeer *et al*, 2010). Hosting of the website allows use of IT resources of the high-resource host country as an in-kind donation. Content of the website is partly determined by needs analysis of participants. Disadvantages are that it may be limited in scope and not a systematic program across all the competencies required by a medical professional group (e.g. HIV/AIDS only, and not the full breadth of Infectious diseases) and may not include audits of patient outcomes.

## Challenges in implementing CPD in low-resource settings

Irrespective of the implementation model there are many challenges in implementing CPD programs in low-resource countries. All programs must be underpinned by ethics and best practice guidelines to avoid burdens for low-resource participating countries (Crump *et al*, 2010). Buy-in by in-country medical professionals and involvement of key opinion leaders are critical for success. Healthcare workers may have a relatively low level of education, and may have difficulty with material presented in non-native language. Cultural barriers may exist in understanding the importance of life-long learning and CPD, and may involve religious and social factors such as in nursing in Bangladesh (Tyer-Viola *et al*, 2013). Resources are constrained, both financial and infrastructure such as hospital computers and medical records. Adequate internet access is important. Medical societies and regulators may be poorly functioning and not able to effectively participate as stakeholders.

High-resource countries may also have challenges in working with low-resource countries. Resources are often constrained, and partnership with not-for-profit international NGOs may be needed to secure financial support. Many programs rely on volunteers to travel at their own expense to low-resource partner countries. Cultural barriers may be present, and exacerbated by lack of common language.

## Opportunities in implementing CPD in low-resource settings

While low-resource countries can gain a functioning CPD program following successful program implementation, high-resource countries can also benefit (Syed *et al*, 2012). High-resource country participants gain intangible benefits such as increased job satisfaction. Health systems in high resource-countries can learn from low resource country innovations including providing services to remote areas, skills substitution, decentralization of management, innovation in mobile phone use and low technology simulation training. The interaction between high and low resource countries can be considered a form of open innovation (Dandonoli, 2013).

## Conclusions

Many low resource countries seek to develop CPD programs for medical professionals. This review identifies 5 general models of implementing programs. Advantages and disadvantages can be identified, however the scientific evidence base for implementing any form of program is weak. Further studies are necessary to determine optimal methods of implementing CPD programs in low-resource countries.

## Figure 1

Figure 1 Basic principles in implementing CPD in developing countries, adapted from Giri et al

Good CPD planning begins with a health education needs assessment.

Principal stakeholders should be involved in formulating the mission and purpose of CPD, as well as the regulatory framework.

Frontline health providers should be involved in system planning and design.

Supervisors and managers should encourage health workers to take advantage of CPD opportunities.

Materials that are of high technical quality but not absorbed or translated into health worker actions are of limited value to the health system.

Appropriate participants must be selected for CPD interventions.

Commercial sponsorship and conflict of interest must be disclosed in the planning of CPD activities.

The best CPD systems are multifaceted and provide different types of formal and informal learning opportunities and support, such as structured courses supported by on-the-job training, mentoring and peer support.

In order to transfer clinical skills, hand-on teaching is more effective than classroom teaching and should be used when feasible.

Adequate internet access is necessary.

## Table 1

Table 1Models for implementing CPD programs in low-resource countries

**Table:** 

**Model**	**Twinning**	**Greenfield**	**Formal program guided by international medical body**	**Directed by regulators**	**Informal, telemedicine-based, guided by international medical body**
Example	CPD program for Cambodian ophthalmologists. Partnership between Cambodian Ophthalmological Society and RANZCO (Mack et, 2017)	CPD program for Lesotho nurses (Moetsana-Poka *et al*, 2014)	CPD program for global ophthalmologists, designed by International Council of Ophthalmology. (www.icoph.org)	MOC examinations in ophthalmology, directed by American Board of Ophthalmology USA in conjunction with the American Medical Association.	Telemedicine support service: for HIV/AIDS healthcare workers in Africa. Hosted by the Institute of Tropical Medicine, Antwerp, Belgium. (Zolfo *et al*, 2006)
Definition	Pairing of one educational organization from a low-resource country with one from a high-resource country to implement a specific medical educational outcome	De novo structured CPD program, designed with stakeholders, including concept of CPD, requirements of program and implementation plan.	Generic structured CPD program designed by international medical body. Intended to be modified prior to implementation by national medical societies prior to implementation.	Structured CPD program designed by national regulatory authorities.	Internet-based program improving education by provision of case studies, frequently-asked questions, and interactive quizzes.

MOC - Maintenance of Certification

## Take Home Messages

## Notes On Contributors

Heather G Mack is an Australian ophthalmologist practicing in medical retina and retinal electrophysiology. She is on the board of the Royal Australian and New Zealand College of Ophthalmologists.

Karl C Golnik is the Neuro-ophthalmologist at the Cincinnati Eye Centre, and Director of Education of the International Council of Ophthalmology.

Neil Murray is an opthalmologist in New Zealand. He is on the board of the Royal Australian and New Zealand College of Ophthalmologists.

Helena P Filipe is a Portugese ophthalmologist practicing in cornea. She is chair of the Continuing Medical Education committee of the International Council of Ophthalmology.

## Bibliography/References

American Medical Association. (2010). *The Physician's Recognition Award and credit system. 2010 revision.* Retrieved from: https://www.ama-assn.org/sites/default/files/media-browser/public/cme/pra-booklet_0.pdf

Archer, J., Pitt, R., Nunn, S., Regan de Bere, S. (2015). *The evidence and options for medical revalidation in the Australian context: Final report.* Collaboration for the Advancement of Medical Education Research and Assessment, Plymouth University Peninsula Schools of Medicine and Dentistry. Melbourne: Medical Board of Australia.

Casebeer, L., Brown, J., Roepke, N., Grimes, C., Henson, B., Palmore, R., Salinas, G.D. (2010). Evidence-based choices of physicians: a comparative analysis of physicians participating in Internet CME and non-participants. *BMC Medical Education*; *10*:42.

https://doi.org/10.1186/1472-6920-10-42

Collins, M., Burn, E., Ritman, D. (2015). A partnership model for the training and professional development of health-care staff in low-resource settings. *Cancer Control*, 79-86. Retrieved from www.cancercontrol.info/wp-content/uploads/2015/07/79-86-Collins.pdf

Cook, D.A., Levinson, A.J., Garside, S., Dupras, D.M., Erwin, P.J., Montori, V.M. (2008) Internet-based learning in the health professions: a meta-analysis. *JAMA, 300*:1181-96.

https://doi.org/10.1001/jama.300.10.1181

Crisp, N. (2007). *Global Health Partnerships. The UK contribution to health in developing countries.* London: DH

Crump, J.A., Sugarman, J. (2010). Working Group on Ethics Guidelines for Global Health Training. *Am J Trop Med Hyg, 83* :1178-1182.

https://doi.org/10.4269/ajtmh.2010.10-0527

Dandonoli, P. (2013). Open innovation as a new paradigm for global collaborations in health. *Global Health, 9*:41.

https://doi.org/10.1186/1744-8603-9-41

Davis, N., Davis, D., Bloch, R. (2008). Continuing medical education: AMEE Education Guide No 35. *Med Teach, 30*:652-66.

https://doi.org/10.1080/01421590802108323

Directive 2013/55/EU of the European Parliament and of the Council of 20 November 2013 amending directive 2005/36/EC on the recognition of professional qualifications and regulation (EU) no 1024/2012 on administrative cooperation through the Internal Market Information System ("the IMI regulation"). 28 Dec 2013. Retrieved from http://eur-lex.europa.eu/LexUriServ/LexUriServ.do?uri=OJ:L:2013:354:0132:0170:en:PDF.

Estrada, C.A., Safford, M.M., Salanitro, A.H., Houston, T.K., Curry, W., Williams, J.H., Allison, J.J. (2011). A web-based diabetes intervention for physician: a cluster-randomized effectiveness trial *. Int J Qual Health Care, 23*: 682-689.

https://doi.org/10.1093/intqhc/mzr053

Filipe, H.P., Silva, E.D., Stulting, A.A., Golnik, K.C. (2014). Continuing professional development: best practices. *Middle East Afr J Ophthalmol* *, 21*: 134-41.

https://doi.org/10.4103/0974-9233.129760

General Medical Council. *Revalidation*. Retrieved from www.gmc-uk.org/doctors/revalidation.asp

Giri, K., Frankel, N., Tulenko, K., Puckett, A., Bailey, R., Ross, H. (2012). *Keeping up to date: Continuing Professional Development for Health Workers in Developing Countries*. Retrieved from Capacity Plus website: https://www.capacityplus.org/technical-brief-6/content/keeping-date-continuing-professional-development-health-workers-developing-countries

Grant, J., Zilling, T. (2015). European regulation of continuing professional development won't improve quality of care. *BMJ, 351*: h5229.

https://doi.org/10.1136/bmj.h5229

Horsfall, S. (2014). *Doctors who commit suicide while under GMC fitness to practice investigation: Internal review.* Retrieved from General Medical Council website: http://www.gmc-uk.org/Internal_review_into_suicide_in_FTP_processes.pdf_59088696

Hudspeth, J., Curry, C.L., Sacks, A., Surena, C. (2015). Continuing Professional Development in Low-Resource Settings: Haiti as Example. *Ann Glob Health, 81*: 255-259.

https://doi.org/10.1016/j.aogh.2015.03.004

Filipe, H.P., Mack, H.G., Mayorga, E.A., Golnik, K.C. (Eds.) (2015). ICO Guide to Effective CPD/CME. Retrieved from International Council of Ophthalmology website: www.icoph.org/resources/339/ICO-Guide-to-Effective-CMECPD-Preview-Edition.html

Lipnick, M., Mijumbi, C., Dubowitz, G., Kaggwa, S., Goetz, L., Mabweijano, J., Ozgediz, D. (2013). Surgery and anesthesia capacity-building in resource-poor settings: description of an ongoing academic partnership in Uganda. *World J Surg* *, 37*: 488-97.

https://doi.org/10.1007/s00268-012-1848-x

Mack, H.G., Meng, N., Parsons, T., Schlenther, G., Murray, N., Hart, R. (2017). Partnering to develop a continuing professional development program in a low-resource setting: Cambodia. *Can J Ophthalmol* in press.

https://doi.org/10.1016/j.jcjo.2016.11.024

Mazmanian, P.E., Davis, D.A. (2002). Continuing medical education and the physician as a learner: guide to the evidence. *JAMA, 288*:1057-60. PMID: 12204068

https://doi.org/10.1001/jama.288.9.1057

McNamara, K. (Ed). (2007). *Improving health, connecting people: The role of ICTs in the health sector of developing countries. A framework paper.* Retrieved from Information for Development Program website: http://www.infodev.org/en/document.84.pdf

Medical Board of Australia. (2016). *Registration standard: Continuing professional development.* Retrieved from Medical Board of Australia website: file:///D:/Downloads/Medical-Board---Registration-standard---Continuing-professional-development---1-October-2016.PDF

Medina, M.T., Munsat, T. (2010). Neurology education in Latin America and the World Federation of Neurology. *J Neurol Sci, 298*: 17-20.

https://doi.org/10.1016/j.jns.2010.07.015

Miller, S.H., Thompson, J.N., Mazmanian, P.E., Aparicio, A., Davis, D.A., Spivey, B.E., Kahn, N.B. (2008). Continuing medical education, professional development, and requirements for medical licensure: a white paper of the Conjoint Committee on Continuing Medical Education. *J Contin Educ Health Prof, 28*: 95-8.

https://doi.org/10.1002/chp.164

Moetsana-Poka, F., Lebaka, M., McCarthy, C.F. (2014). Developing a continuing professional development program to improve nursing practice in Lesotho. *Afr J Midwifery Womens Health, 8 (Suppl 2)*: 10-13. PMID: 27053950

https://doi.org/10.12968/ajmw.2014.8.Sup2.10

Royal Australian and New Zealand College of Ophthalmologists. (2016). *Continuing professional development handbook 2016.* Retrieved from Royal Australian and New Zealand College of Ophthalmologist website: file:///D:/Downloads/cpd%20handbook.pdf

Syed, S.B., Dadwal, V., Rutter, P., Storr, J., Hightower, J.D., Gooden, R., Pittet, D. (2012). Developed-developing country partnerships: benefits to developed countries? *Global Health, 8*: 17.

https://doi.org/10.1186/1744-8603-8-17

Teasdale, G.M., Council of the Society of British Neurological Surgeons. (2002). Learning from Bristol: report of the public inquiry into children's heart surgery at Bristol Royal Infirmary 1984-1995. *Br J Neurosurg, 16*: 211-216. PMID: 12201391

https://doi.org/10.1080/02688690220148815

Tyer-Viola, L.A., Timmreck, E., Bhavani, G. (2013). Implementation of a Continuing Education Model for Nurses in Bangladesh. *J Contin Educ Nurs, 44*: 470-476.

https://doi.org/10.3928/00220124-20130816-07

World Federation for Medical Education. (2009). *Details of planned collaborative activities with WHO for 2010-2012.* Retrieved from World Federation for Medical Education website: http://wfme.org/about/other-wfme-partners/who

Yoon HB, Shin JS, Bouphavanh K, Kang YM (2016). Evaluation of a continuing professional development training program for physicians and physician assistants in hospitals in Laos based on the Kirkpatrick model. *J Educ Eval Health Prof, 13*:21.

https://doi.org/10.3352/jeehp.2016.13.21

Zagorski Z. (2006). *Principles and Guidelines of a Curriculum for Continuing Medical Education in Ophthalmology*. Retrieved from International Council of Ophthalmology website: http://www.icoph.org/dynamic/attachments/resources/icocurriccme.pdf

*Zolfo,* M., Lynen, L., *Dierckx, *J., Colebunders, R. (2006). Remote consultations and HIV/IADS continuing education in low-resource settings. *Int J Med Inform, 75*: 633-637.

https://doi.org/10.1016/j.ijmedinf.2006.03.002

## Appendices

## Declarations

The author has declared that there are no conflicts of interest.

