# Supplementary material for: Models for implementing continuing professional development programs in low-resource countries
Source: MedEdPublish (2016). 2017 Feb 1;6:18. [Version 1] doi: 10.15694/mep.2017.000018 (PMC10885220; doi:10.15694/mep.2017.000018)
Supplement: Peer review history

# Reviewer report: Reviewer response for version 1

**DOI:** 10.21956/mep.19434.r28591

**Published:** 2017-02-11

**Authors:** Michelle McLean

**Affiliations:** 1 Bond University

This review has been migrated. The reviewer awarded 4 stars out of 5 This is one of the first papers in the Medical Education in Difficult Circumstances to offer solutions, which helps us with closing the loop, It is easy to identify problems, but finding solutions is always the problems. The summary you have provided is a useful start towards addressing the problem. As has been pointed out by some of my colleagues' reviews, these models involve outside parties and so there is always the question of sustainability. Developing home-grown capacity is therefore important,

Reviewer Expertise:

NA

No decision status is available

# Reviewer report: Reviewer response for version 1

**DOI:** 10.21956/mep.19434.r28590

**Published:** 2017-02-04

**Authors:** Trudie Roberts

**Affiliations:** 1 Leeds Institute of Medical Education

This review has been migrated. The reviewer awarded 3 stars out of 5 I really enjoyed reading this and think it was an interesting paper for all those involved in Faculty development not only in low resource countries. As others have commented it would be good to know about sustainability and the appropriateness of the model for particular situations and even if at different times in the evolution of an institution different models might be appropriate. Perhaps staff involved in some of the examples given could write about their own programme and directly address the issue of sustainability

Reviewer Expertise:

NA

No decision status is available

# Reviewer report: Reviewer response for version 1

**DOI:** 10.21956/mep.19434.r28588

**Published:** 2017-02-02

**Authors:** Trevor Gibbs

**Affiliations:** 1 AMEE

This review has been migrated. The reviewer awarded 4 stars out of 5 I enjoyed reading this paper and thought that it gave some very good ideas to those involved in developing CPD programmes. The more one travels around reviewing medical educational programmes or reads about medical education in other countries, it is still very surprising to detect the weakness in many CPD programmes, specifically about purpose. Many working practitioners see CPD far too often as a burden that should be done as quickly as possible and with as little effort as possible. The paper presented, very clearly, five very acceptable approaches, all of which would work and from which countries could choose. However, I think I would have liked to have seen a little further clarity on if one approach was better than another and also a little bit more on which brings out the motivation in the participant. Far too often CPD programmes are purposeful in only one particular area and their translation into practice far from obvious- participants in programmes need to know that their work in such programmes will have outcomes, will reward them in a clinical sense and reward their community in medico-social sense,I do feel that this paper will help those those involved in CPD programming but I hope that it can be expanded into addressing some of these motivational issues

Reviewer Expertise:

NA

No decision status is available

# Reviewer report: Reviewer response for version 1

**DOI:** 10.21956/mep.19434.r28589

**Published:** 2017-02-02

**Authors:** Richard Hays

**Affiliations:** 1 James Cook University

This review has been migrated. The reviewer awarded 3 stars out of 5 This is an interesting paper that describes clearly five different models of helping low resource countries enhance health care through CPD programs. What may be missing is an analysis of what works best, although measuring success can be difficult. My observation of several cases of support - hence only weak evidence - is that sustainability is enhanced through longer term arrangements and a clear emphasis from the outset on transfer of skills and knowledge (sometimes also equipment) to people in the low resource settings. While it can be rewarding for all concerned when an outsider helps during visits, caution is needed to avoid dependency on visitors and distant institutions, even when this is not the intention. There is a risk that locals are trained to the point where they feel they have to leave to pursue certain careers that are not sustainable unless the resources are provided locally. I would welcome the authors thoughts on the issue of sustainability.

Reviewer Expertise:

NA

No decision status is available
